# Enhancement of antitumor immune response by radiation therapy combined with dual immune checkpoint inhibitor in a metastatic model of HER2-positive murine tumor

**DOI:** 10.1007/s11604-022-01303-z

**Published:** 2022-06-28

**Authors:** Sayaka Misaki, Satoshi Murata, Miyuki Shimoji, Takayasu Iwai, Andreas Michael Sihombing, Ken Aoki, Yutaka Takahashi, Yoshiyuki Watanabe

**Affiliations:** 1 Department of Radiology, Shiga University of Medical Science, Seta Tsukinowa-Cho, Otsu, Shiga 520-2192 Japan; 2 Cancer Center, Shiga University of Medical Science Hospital, Seta Tsukinowa-Cho, Otsu, Shiga 520-2192 Japan; 3 Department of Surgery, Shiga University of Medical Science, Seta Tsukinowa-Cho, Otsu, Shiga 520-2192 Japan; 4 Department of Medical Physics and Engineering, Osaka University Graduate School of Medicine, 1-7, Yamada-oka, Suita, Osaka 565-0871 Japan

**Keywords:** Radioimmunotherapy, Tumor antigen-specific CD8-positive T-lymphocyte, Cytotoxic T-lymphocyte, Immune checkpoint inhibitor, Abscopal effect

## Abstract

**Purpose:**

Treatments for metastatic human epidermal growth factor receptor 2 (HER2)-positive tumors are improving but remain inadequate. We investigated activating antitumor immune response by combining radiation therapy with immune checkpoint inhibitors using mouse tumors overexpressing HER2, a pivotal driver oncogenic antigen, to develop new immunotherapies for metastatic HER2-positive tumors.

**Materials and methods:**

NT2.5 cells were inoculated into the two mammary fat pads of FVB/N mice, which were divided into four groups: no treatment (Non), anti-PD-1 and anti-CTLA4 antibodies (P1C4), irradiation of the large tumor (Rad), and combination (R + P1C4) groups. Tumor growth, immunostaining of tumor-infiltrating lymphocytes, and the proportion of HER2-tumor antigen-specific CD8-positive T cells in the spleen and tumor-infiltrating lymphocytes were analyzed.

**Results:**

In the Rad group, unirradiated and irradiated tumors shrank after treatment. Besides the directly irradiated tumors, the unirradiated tumors in the R + P1C4 group shrank the most. In the unirradiated tumors, CD8-positive T cells and FOXP3-positive T cells accumulated significantly more in the R + P1C4 group than in the P1C4 and the Rad groups (all *p* < 0.001). CD4-positive helper T cells accumulated significantly more in the R + P1C4 group than in the Rad group (*p* < 0.05), but this was not significantly different from the P1C4 group. HER2-specific CD8-positive T cells in the spleen and tumor-infiltrating lymphocytes were significantly increased in the R + P1C4 group compared to the P1C4 and Rad groups (all *p* < 0.0001).

**Conclusion:**

Irradiation of HER2-positive tumors induced an antitumor immune effect against the unirradiated tumor, which was enhanced by the combined use of immune checkpoint inhibitors and was mediated by enhanced recruitment of HER2-tumor antigen-specific cytotoxic T lymphocytes at the tumor site in an HER2-positive mouse tumor model. Harnessing the distant antitumor immune response induced by the combination of radiation therapy and immune checkpoint inhibitors could be a promising treatment strategy for metastatic HER2-positive tumors.

## Introduction

Radiation therapy (RT) is a local treatment for malignant tumors that was thought to suppress immunity [1]. However, in 1979, the role of T cells in the effects of RT on local tumor control was shown in a murine fibrosarcoma model [2], and many studies have reported their immunogenic effect [3–5]. Irradiation can elicit tumor antigen-specific cellular immunity [3, 4]. Additionally, the RT-induced immune-mediated antitumor effect causes tumor shrinkage outside the irradiated field, termed the abscopal effect [5].

The combination of an immune checkpoint inhibitor (ICI) and RT was first used in a mouse breast cancer model, resulting in suppressed metastasis [6]. Clinical and basic research on the timing of concomitant use with immunomodulators is ongoing [7].

Human epidermal growth factor receptor 2 (HER2) is a driver gene of cell proliferation [8]. HER2 overexpression promotes breast cancer carcinogenesis [8]. Furthermore, HER2 expression is a poor prognostic factor for breast cancer [9]. As HER2 is important for cancer cell growth, it is attracting attention as an immunogenic tumor antigen [10].

HER2-positive tumor survival has been improved by new anti-HER2 drugs. However, nearly 20% of early stage HER2-positive breast cancers recur [11], and recurrence or metastasis remains intractable. To overcome this, we investigated the local RT-induced distant antitumor effect of ICIs in HER2-positive metastatic tumor model mice. We also investigated the effects of irradiation and ICIs on cellular immunity by examining the expression of HER2-antigen-specific cytotoxic T lymphocytes (CTLs) in the spleen and unirradiated tumors. If the distant antitumor effect is effective in treating HER2-positive tumors and its immunological mechanism is elucidated, it may lead to novel immunotherapies for metastatic HER2-positive tumors.

## Materials and methods

### Mice

FVB mice were purchased from CLEA Japan (Tokyo, Japan) and mated. We used 6–10-week-old female mice. The animals were housed under pathogen-free conditions at Shiga University of Medical Science university. Experiments were performed according to the institutional and national guidelines for the care and use of animals and institution-approved protocols were followed.

### Cell lines

NT2.5, a cell line derived from HER2/neu (rat) transgenic mouse mammary tumor, was grown in defined breast medium and maintained at 37 °C in 5% CO_2_, as previously described [12]. T2D^q^ cells were created by transfecting T2 cells lacking the MHC class 1 antigen processing-related transporter with the D^q^ gene encoding the MHC class 1 molecule of RNEU_420-429_, an epitope of the neu antigen [12].

### Peptides and antibodies

RNEU_420-429_ (PDSLRDLSVF), the immunodominant peptide of HER2/neu for MHC class I, and NP_118-126_ (RPQASGVYM), a peptide derived from a nuclear protein as a control, were obtained from Hokkaido System Science (Sapporo, Japan). An APC-anti-mouse CD8a monoclonal antibody (mAb), PE-anti-mouse IFN-γ mAb, and Fixable Viability Stain 520 were obtained from BD Biosciences (Franklin Lakes, NJ, USA). A therapeutic anti-PD-1 mAb (clone: RMP1-14) and anti-CTLA-4 mAb (clone: 9H10) were obtained from BioXcell (Lebanon, NH, USA). IgG from rat serum, administered as a therapeutic control, was obtained from Sigma-Aldrich (St. Louis, MO, USA). The antibodies (Abs) used for immunostaining were as follows: anti-CD8 mAb (ab209775, 1:2000, Abcam, Cambridge, United Kingdom), anti-CD4 mAb (ab183685, 1:1000, Abcam), anti-FOXP3 Ab (polyclonal, NB100-39002, 1:800, Novus Biologicals, Centennial, CO, USA), and Histofine Simple Stain Mouse MAX-PO (Nichirei Biosciences, Tokyo, Japan).

### Tumor cell challenge and treatment

FVB mice were injected subcutaneously with 5 × 10^6^ cells in the second mammary fat pad from the bottom bilaterally. Tumor diameters were measured orthogonally with a digital caliper every 2–4 days, and tumor sizes were calculated as length × width (mm^2^). When the tumor reached 25–35 mm^2^ or more (17–28 days post-inoculation), mice were randomly assigned to two or four groups. For the two groups, mice were assigned to a no-treatment group (Non, *n* = 4) and a group that received RT for the larger tumor (Rad, *n* = 4).

**Fig. 1:**
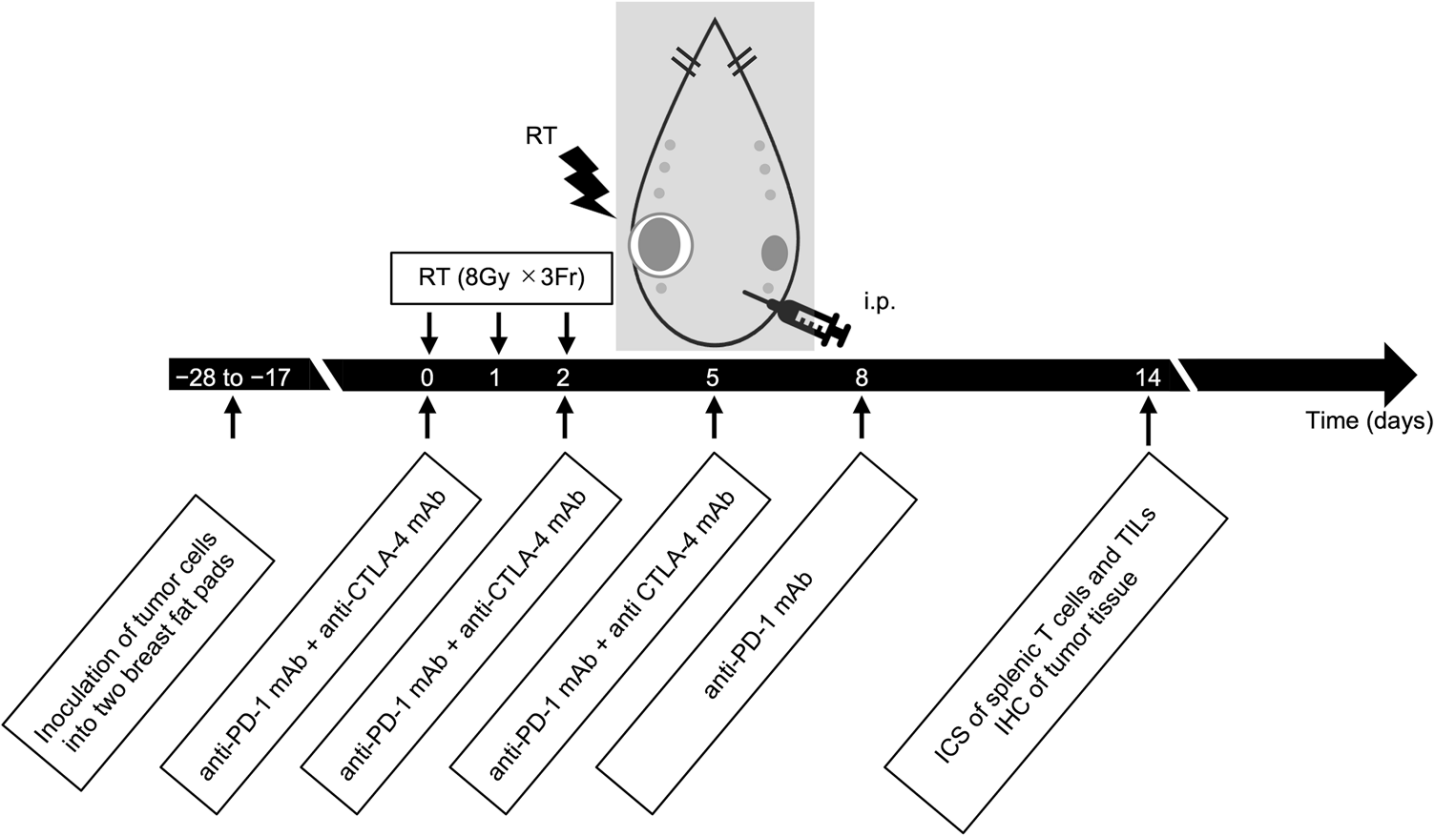
The experiment schedule. Tumor cells (5 × 10^6^) overexpressing HER2/neu were inoculated bilaterally into the mammary fat pads of female FVB mice. When tumors reached approximately 30–100 mm^2^, treatment commenced in four groups: no treatment (Non), anti-PD-1 and anti-CTLA-4 antibodies (P1C4), irradiation of the large tumor (Rad), and combination (R + P1C4) groups. Radiation therapy and administration of immune checkpoint inhibitors (ICIs) were performed as shown in the figure. ICIs were administered at 150 μg each

The four groups were: the Non group (*n* = 4), a group administered PD-1 and CTLA-4 mAbs (P1C4 group, *n* = 6), Rad group (*n* = 6), and a combined group administered mAbs and RT (R + P1C4 group, *n* = 6). For ICIs, anti-PD-1 and anti-CTLA4 mAbs were administered on days 0, 2, and 5 after the start of treatment and only the anti-PD-1 mAb on day 8. Rat IgG was administered as a control in the Non and Rad groups. Each antibody was administered intraperitoneally (150 µg). During RT, mice were anaesthetized by an intraperitoneal injection of 5 mg/kg of ketamine hydrochloride (Ketalar, Daiichi Sankyo, Tokyo, Japan) and 1.0 mg/kg of medetomidine hydrochloride (Domitor, Zonoac, Fukushima, Japan). The larger of the two tumors was irradiated with three fractions of 8 Gy on days 0, 1, and 2 using the Xstrahl RS320 X-ray irradiator (Camberley, United Kingdom) at 150 kVp, 20 mA with a 0.5 mm Al and 0.1 mm Cu filter. During irradiation, mice were laid supine in an acrylic case, and the whole body was shielded with a 5 mm-thick lead plate. The tumor on the irradiated side was placed outside the shield through a 1 cm hole (Fig. 1).

### HER2/neu tumor antigen-specific CD8-positive T-cell assay of splenocytes

Spleens of all mice were harvested 14 days after beginning treatment. Splenic T cells were purified from splenocytes using a nylon wool column after treatment with Red Blood Cell Lysis Solution (Miltenyi Biotec, Bergisch Gladbach, Germany). Purified T cells were restimulated overnight with T2D^q^ cells pulsed with RNEU_420-429_ or NP_118-126_. Intracellular cytokine staining (ICS) to assess IFN-γ production by CD8-positive T cells in response to RNEU was performed by flow cytometry according to the Cytofix/Cytoperm with the Golgistop procedure (BD Biosciences) as described previously [13].

### Tumor-infiltrating T-cell assay

Tumors were removed 14 days after beginning treatment. Tumors were diced and digested with collagenase IV and 0.1% hyaluronidase using a bioshaker for 2 h at 37 °C. The centrifuged debris was trypsinized, and the cell pellets were cultured for 2 h at 37 °C under 5% CO_2_ to remove the adherent NT2.5-derived tumor cells. Non-adherent cells were collected and filtered with a cell strainer (Easy strainer 70 μM, Greiner Bio-one, Oberosterreich, Austria). Finally, tumor-infiltrating lymphocytes (TILs) were prepared from the flow-through fraction of a nylon wool column. HER2/neu tumor antigen-specific CD8-positive T cells were examined using the above assay with an Fcγ receptor blocker (Miltenyi Biotec) before staining with the CD8a mAb to avoid non-specific binding.

### Flow cytometry

Flow cytometry data were collated using BD FACS Calibur (BD Biosciences) and analyzed using FlowJo v10 software (BD Biosciences).

### Immunohistochemical staining and analysis

Tumors were removed 14 days after beginning treatment and were fixed in 4% paraformaldehyde phosphate buffer solution (Nacalai Tesque, Kyoto, Japan) for 24 h, transferred to 70% ethanol, and processed into paraffin blocks. The blocks were sectioned at 4 μm, followed by deparaffinization and antigen retrieval in 10 mM sodium citrate buffer (pH 6) at 98 °C for 45 min. Immunohistochemistry (IHC) was then performed as follows: quenching with 3% hydrogen peroxide in methanol for 13 min, blocking with Histofine Blocking Reagent A (Nichirei Biosciences) for 45 min, primary antibody incubation overnight at 4 °C, blocking with Histofine Blocking Reagent B (Nichirei Biosciences) for 20 min, secondary antibody incubation for a half hour, and 3,3′-diaminobenzidine (Nichirei Biosciences) treatment for 5 min. The sections were counterstained with hematoxylin.

IHC was performed on three specimens from each group, and the number of immunostained cells was counted. Two independent observers each counted positive cells from all specimens in five non-overlapping high-power fields (400×). The average number of positive cells in each group was calculated. We selected fields of view with many positive cells in and around the tumor. For CD4/8-positive cells, those with a clearly stained cell membrane were counted, while for FOXP3, those with a deeply stained nucleus were counted. The number of FOXP3-positive cells was subtracted from the number of CD4-positive cells to yield the number of CD4-positive helper T cells.

### Statistical analysis

All data are presented as the mean ± standard deviation (SD). One-way analysis of variance (ANOVA) was performed to assess significant differences among three or four groups in tumor size, the proportion of tumor antigen-specific CD8-positive T cells in TILs and splenocytes, and the number of immunostained cells. If there was a significant difference, a Bonferroni correction was performed as a post hoc test. The proportion of tumor antigen-specific CD8-positive T cells in splenocytes between the Non and Rad groups was assessed by a two-tailed Student’s t test. The reliability between examiners in the IHC experiment was examined using intraclass correlation coefficients (2, 1). All statistical analyses were performed with EZR version 1.54 (Saitama Medical Center, Jichi Medical University, Saitama, Japan) [14], a graphical user interface for R. More precisely, a modified version of R commander (version 1.6–3) added statistical functions frequently used in biostatistics. Statistical significance was set at* p* < 0.05.

## Results

### Induction of distant antitumor effect by irradiation

**Fig. 2:**
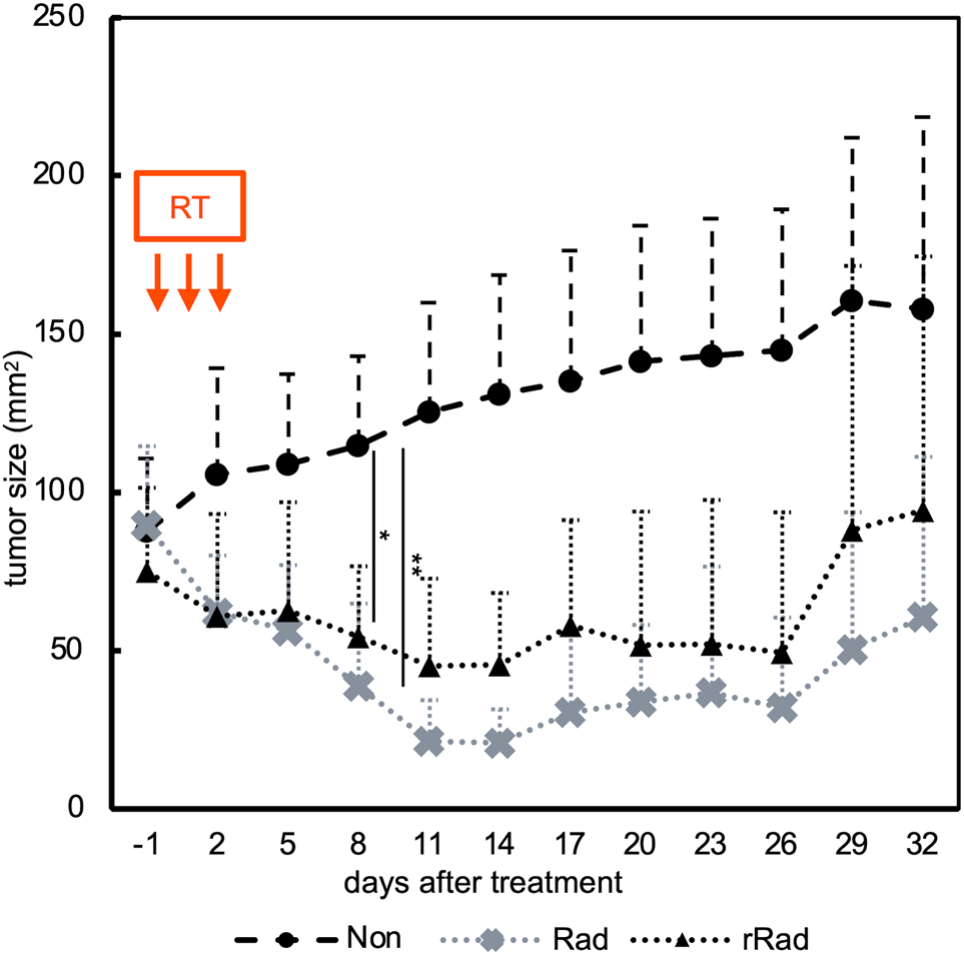
Tumor growth curve in the Non and Rad groups. The orthogonal tumor diameter (length and width) was measured every 3 days, and tumor size was calculated as length × width (mm^2^). The graph shows the change in tumor size between the Non and Rad groups. Mean tumor sizes ± SD of bilateral tumors in the Non group (*n* = 8), Rad (*n* = 4) and unirradiated tumors in the Rad group (r-Rad, *n* = 4) are shown. *p  < 0.05, **p  < 0.01

To investigate whether RT could induce the distant antitumor effect, or the so-called abscopal effect, in HER2-positive cancer-bearing mice, we evaluated tumor sizes bilaterally in the Rad and Non groups. Tumors grew rapidly in the Non group, while in the Rad group, both unirradiated and irradiated tumors exhibited growth inhibition after beginning treatment (Fig. 2). This finding indicates that the distant antitumor effect was induced by RT in HER2/neu-expressing tumors.

Spleens were removed 14 d after beginning treatment in the Non and Rad groups to confirm whether a systemic tumor antigen-specific immune response was induced. Splenic T cells were restimulated with T2D^q^ cells pulsed with RNEU or a control peptide. Subsequently, the proportion of CD8-positive T cells secreting IFN-γ was determined by ICS. The proportion of RNEU-specific CD8-positive T cells in the RT group was significantly increased compared to that in the Non group (*p* = 0.046; No*n* = 0.068%, Rad = 0.12%).

### Enhancement of RT-induced distant antitumor effect by ICIs

**Fig. 3:**
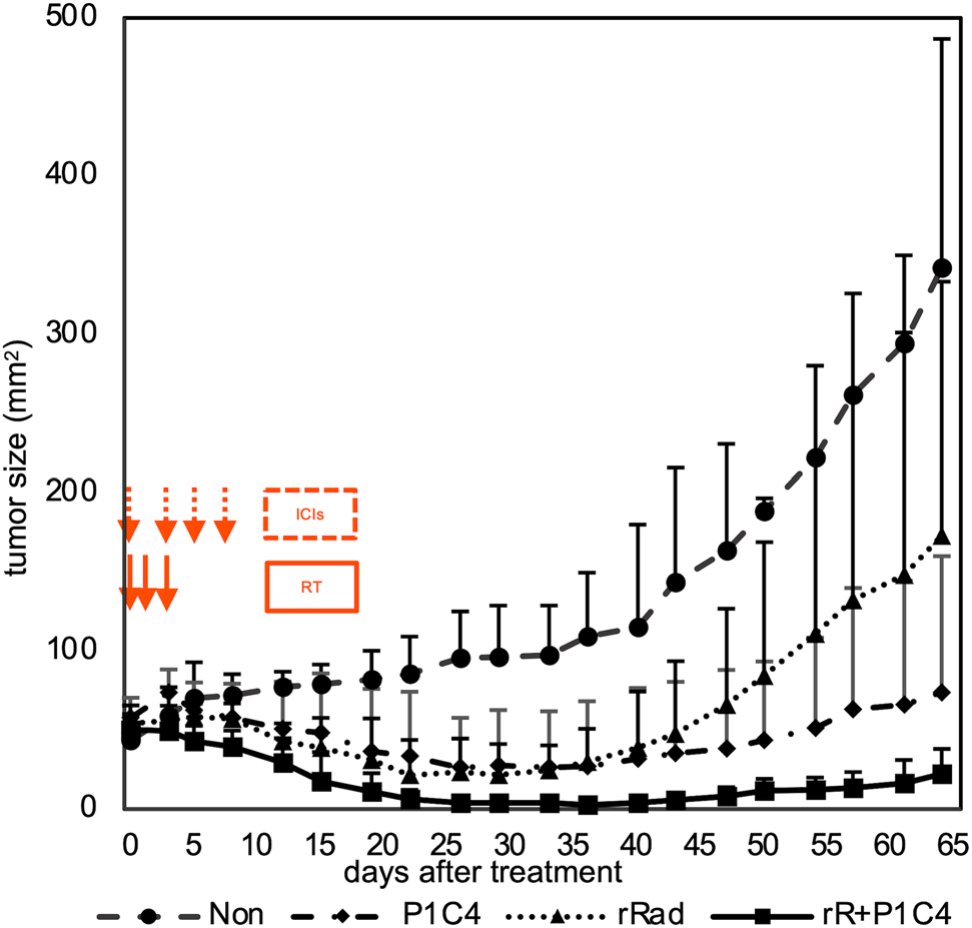
Tumor growth curve in Non, P1C4, Rad, and RP1C4 groups. The orthogonal tumor diameter (length and width) was measured every 2–4 days, and tumor size was calculated as length × width (mm^2^). The graph shows the size changes in the following four groups: no treatment (Non, *n* = 4), anti-PD-1 and anti-CTLA-4 antibodies (P1C4, *n* = 6), irradiation of the large tumor (Rad, *n* = 6), or combination (R + P1C4, *n* = 6) groups. The mean tumor size ± SD of small tumors in the Non (*n* = 4) and P1C4 (*n* = 6) groups and unirradiated tumors in the Rad (r-Rad, *n* = 6) and R + P1C4 (rR + P1C4, *n* = 6) groups are shown

The growth of unirradiated tumors among the four groups (Non, *n* = 4, Rad, P1C4, and R + P1C4, *n* = 6) is shown in Fig. 3. Tumors in the Non group continued to grow, while tumors in other groups exhibited substantially suppressed growth post-treatment. Tumors in the P1C4 group and unirradiated tumors in the Rad group initially showed similar growth inhibition, but the Rad group grew rapidly after 40 d treatment. Unirradiated tumors in the R + P1C4 group showed the most growth suppression, but no mice showed complete response. These findings indicate that ICIs enhance the RT-induced distant antitumor effect.

### Enhancement of RT-induced antitumor immunity by ICIs

**Fig. 4:**
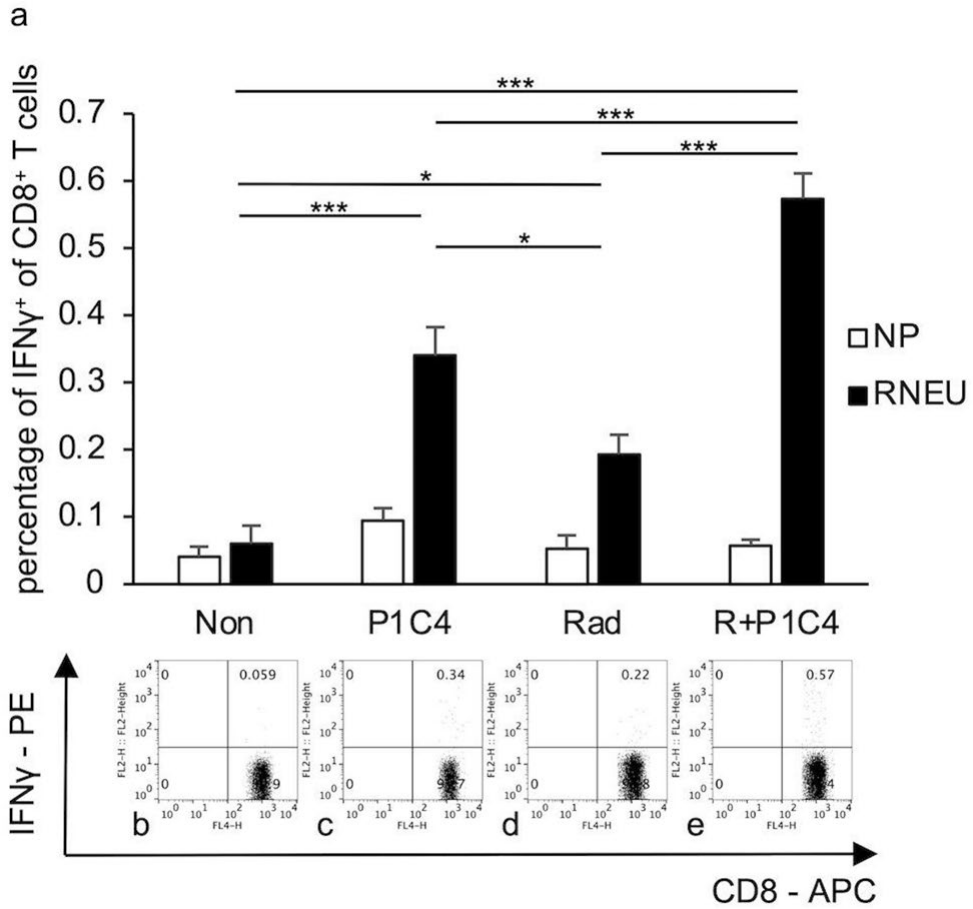
Frequency of HER2/neu-specific effector CD8-positive T cells in splenocytes. Spleens of mice from four groups (Non, P1C4, Rad and R + P1C4) were removed 14 days after beginning treatment. Splenic T cells were determined by the percentage of CD8-positive T cells expressing IFN-γ when exposed to RNEU (immunodominant against the tumor antigen neu) or a control peptide nuclear protein (NP) by intracellular staining. Upper row **a** representative data for the proportion of HER2/neu-specific effector CD8-positive T cells in each group from triplicate experiments. Error bars show three average SD values. Bottom row (**b**–**e**): dot plot. *p < 0.05, **p < 0.01, ***p  < 0.001

Figure 4 shows the results of inducing systemic tumor antigen-specific immune response in the spleen using RT with ICIs. The proportion of RNEU-specific CD8-positive T cells was significantly higher in the R + P1C4 group than in the P1C4 (*p* = 0.00072) and Rad (*p* = 0.000014) groups.

**Fig. 5:**
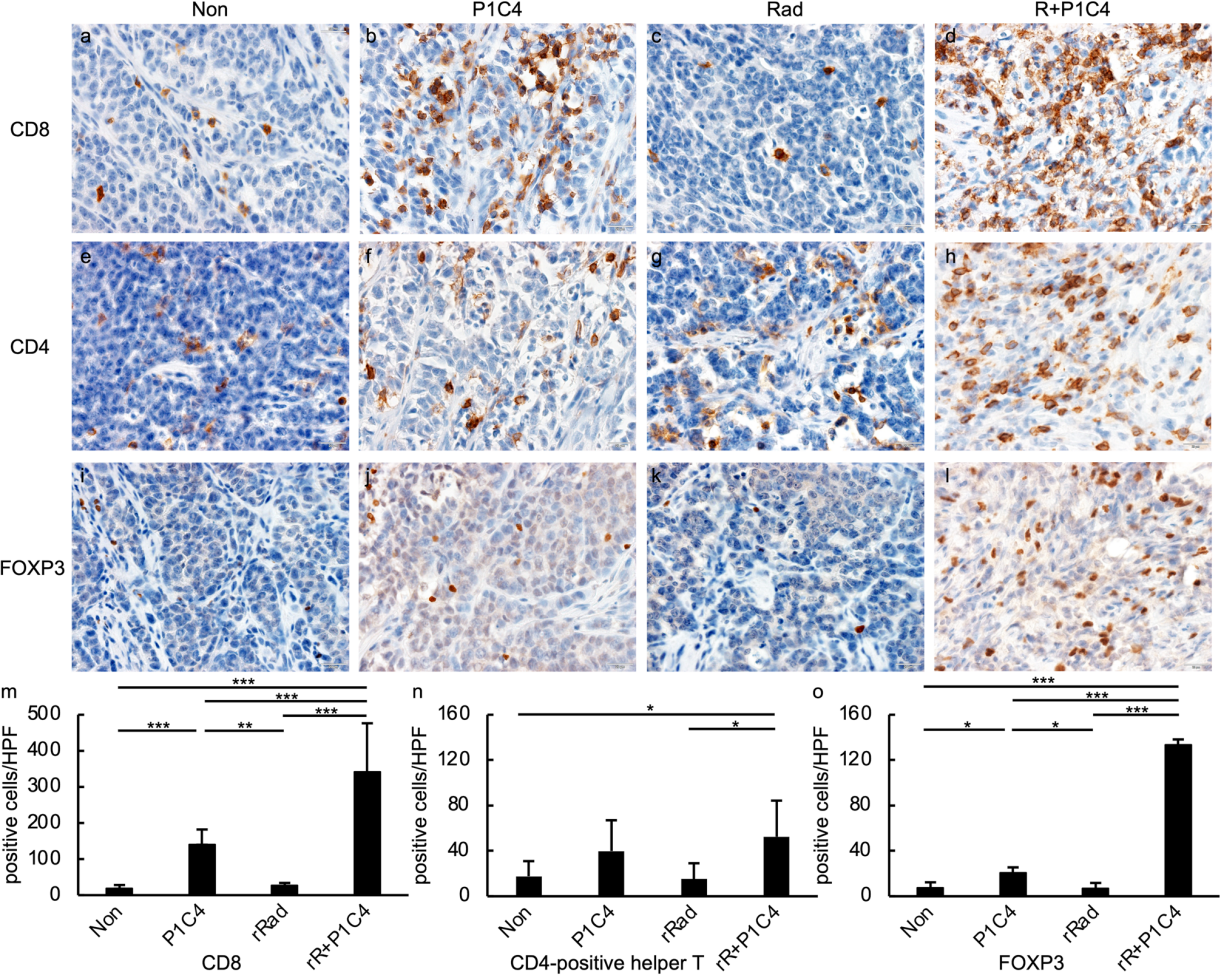
Expression of CD8, CD4, and FOXP3 in TILs of unirradiated tumors. Tumors of mice from four groups (Non, P1C4, Rad, and R + P1C4) were removed 14 days after beginning treatment. Immunohistochemical staining (CD8, CD4, and FOXP3) was performed. The number of CD4-positive helper T cells was calculated by subtracting the number of FOXP3-positive cells from the number of CD4-positive cells. Upper row: **a**–**l** typical images of each immunostaining. Positive cells are shown as brown. Lower graph: **m**–**o** the average number of positive cells in the three tumors. The reliability between the observers was ICC (2,1) = 0.84. Error bars show average SD values. *p  < 0.05, **p  < 0.01, ***p  < 0.001

Whether antitumor immunity was induced locally in the tumor was determined by IHC (Fig. 5). Unirradiated tumors in the Rad group did not exhibit more TILs compared to that in the Non group. Tumors in the P1C4 group had significantly increased number of CD8-positive cells compared to the Non group (*p* = 0.0061). Unirradiated tumors in the R + P1C4 group had significantly increased number of CD8-positive cells compared to the Rad group (*p* < 0.00001) and in the P1C4 group (*p* = 0.0086). In addition, unirradiated tumors in the R + P1C4 group showed higher infiltration of CD4-positive helper T cells and FOXP3-positive cells than that observed in the Rad group (*p* < 0.05, *p* < 0.001, respectively) and in the P1C4 group (*p* = 0.77, *p* < 0.001, respectively).

**Fig. 6:**
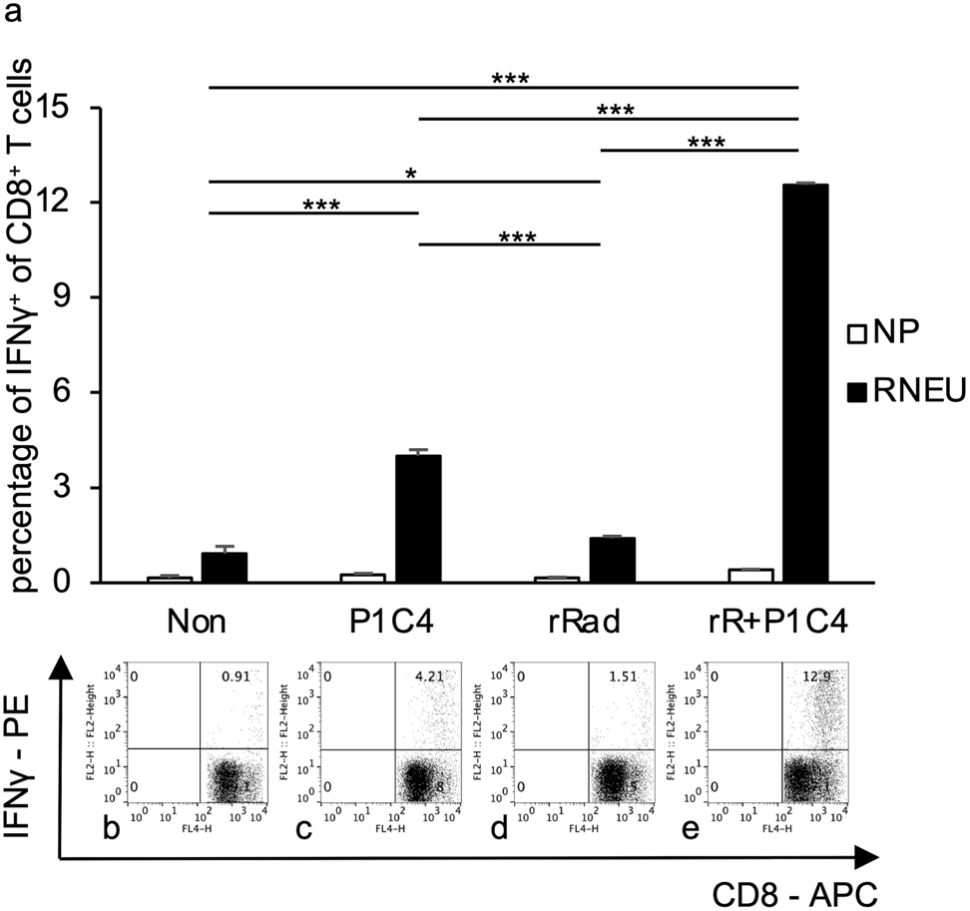
Frequency of HER2/neu-specific effector CD8-positive tumor-infiltrating T cells of unirradiated tumors. Tumors of mice from four groups (Non, P1C4, Rad, and R + P1C4) were removed 14 days after beginning treatment. Tumor-infiltrating lymphocytes (TILs) were purified and the percentage of CD8-positive T cells expressing IFN-γ when exposed to RNEU (immunodominant against the tumor antigen neu) or the irrelevant target nuclear protein (NP) by intracellular staining were determined. Upper row: **a** representative data for the proportion of HER2/neu-specific effector CD8-positive T cells in each group of TILs (triplicate experiments). In the groups that included RT, data on the unirradiated side are shown. Error bars show three average SD values. Bottom row: **b**–**e**: a typical dot plot. *p  < 0.05, **p  < 0.01, ***p  < 0.001

Figure 6 shows the results of tumor antigen-specific immune response induction in TILs. Unirradiated tumors in the Rad group showed significantly more RNEU-specific CD8-positive T cells of the TILs compared to those in the Non group (*p* = 0.027). Additionally, unirradiated tumors in the R + P1C4 group demonstrated significantly increased percentages of RNEU-specific CD8-positive T cells in TILs compared to unirradiated tumors in the Rad (*p* < 0.00001) or P1C4 (*p* < 0.00001) groups.

## Discussion

We established a model to induce a local RT-induced distant antitumor effect outside the irradiated field using tumor cells overexpressing HER2/neu, an essential tumor progression driver. This study had three main findings. First, RT alone induced the distant antitumor effect in HER2/neu-expressing tumors. Second, RT to HER2/neu-positive tumors produced tumor antigen-specific CTLs, which may underlie the distant antitumor effect. Third, addition of dual ICI to RT increased the accumulation of tumor antigen-specific CTLs in the spleen and at the tumor site, thereby enhancing the distant antitumor effect.

Dewan et al. [15] showed that induction of tumor antigen-specific CD8-positive T cells by RT caused tumor shrinkage outside the irradiated field. Irradiation may release damage-associated molecular patterns and other substances, activating dendritic cells, thereby inducing antigen-specific CD8-positive T cells [16]. We demonstrated that RT against HER2/neu-expressing tumors induced tumor antigen-specific CD8-positive T cells in the spleen and caused these cells to accumulate in the tumor. Furthermore, CD8-positive T cells produced cytokines in response to the HER2/neu tumor antigen. Therefore, these tumor antigen-specific immune mechanisms may contribute substantially to the distant antitumor effect outside the irradiated field.

In clinical practice, tumor shrinkage outside the irradiated field is rarely induced by RT alone; even in animal models, the distant antitumor effects of RT alone are weak. However, Demaria et al. [6] reported that co-administration of an anti-CTLA4 mAb with RT of 4T1 mouse breast cancer both enhanced the therapeutic effect of irradiated local tumors and suppressed metastasis. RT with immunomodulators, such as an ICI, enhances the distant antitumor effect [15, 17]. Victor et al. [17] reported that the combination of anti-PD-L1 and anti-CTLA4 mAbs had a higher antitumor effect than the use of an ICI alone. Our preliminary study, which used the same tumor model as this study, showed that the combination of RT and dual ICI was more effective than the combination of RT and an ICI monotherapy (data not shown). Accordingly, we utilized the combination of RT and dual ICI in this investigation. Here, dual ICI combined with RT further suppressed HER2-positive tumor growth on the unirradiated side compared to RT alone, indicating an enhanced the RT-induced antitumor immune effect. The combination of RT and dual ICI showed increased HER2-specific CD8-positive T cells in the spleen and increased TILs. Among the TILs, HER2-specific CD8-positive T cells had an increased proportion. The HER2/neu tumor antigen is essential for the growth of HER2-positive tumors such as human breast cancer, as well as the NT2.5 cells used in this study. The findings in this study suggest that the combination of RT and ICIs may improve the distant antitumor effect away from the irradiation field by inducing systemic and local enhancement of the specific immune response against essential tumor antigens.

ICI is attracting attention as a therapeutic agent for HER2-positive tumors. Following a clinical trial for HER2-positive gastric cancer and gastro-esophageal junction cancer, the Food and Drug Administration approved the addition of an anti-PD-1 mAb to a conventional first-line treatment with combination chemotherapy and an anti-HER2 mAb [18]. In contrast, clinical trials of anti-HER2 and anti-PD-1 mAbs for high-risk HER2-positive breast cancer were discontinued due to poor interim results [19]. Although treatment of HER2-positive tumors with RT and an ICI remains uncommon, RT combined with anti-CTLA-4 and anti-HER2 mAbs increased the disease control rate after 12 weeks in patients with HER2-positive breast cancer and brain metastases [20]. These findings suggest that successful treatment of ICIs requires tumor antigen release, an important part of the cancer-immunity cycle [21], which is elicited by cytotoxic treatments such as chemotherapy or irradiation for HER2-positive tumors. We have shown that the combination of dual ICI and RT, rather than dual ICI alone, enhances the generation of HER2 antigen-specific CTLs and the distant antitumor effect in HER2-overexpressing tumors, indicating that RT is essential for enhancing the function of ICIs. Taken together, the combination of tumor irradiation and systemic dual ICI administration is promising for the treatment of distant metastatic HER2-positive tumors.

In this study, cellular immunity was induced in tumors treated with RT and ICIs; however, simultaneously, FOXP3-positive regulatory T cells (Tregs), which suppress the cellular immunity, were also accumulated in the tumor. The presence of TILs is positively associated with improved lymph-node status and prognosis [22], and that the presence of CD8-positive CTLs in breast cancer is associated with good outcomes [23, 24]. In contrast, the presence of FOXP3-positive Tregs in breast cancer has been paradoxically reported to be associated with both reduced and improved survival [25]. In addition, a statistically significant positive correlation was reported between CD8-positive and FOXP3-positive cell infiltration in TILs [25]. Furthermore, the infiltration of CD8-positive cells and a high CD8/FOXP3 ratio are associated with a good prognosis in breast cancer [26]. These findings suggest that the increase in Tregs may be an inhibitory response to the increased cellular immunity induced by combination therapy with RT and ICIs.

One strategy to improve this treatment is to determine the optimal timing of ICIs administration to enhance the distant antitumor effect outside the irradiated field. Here, the anti-CTLA-4 mAb was administered concurrently with the start of RT to function during the priming phase of T-cell activation, and the anti-PD-1 mAb was administered to work during the T-cell effector phase. However, a previous study reported that the survival rate was higher when an anti-CTLA-4 blockade was used before RT [25] as a result of Tregs depletion. Therefore, the use of the anti-CTLA4 mAb prior to RT may suppress Tregs infiltration into the tumors mediated by the combination of RT and ICIs, leading to improved therapeutic effects. Further studies must determine the optimal timing of administration.

Another strategy is to find an efficient drug combination to increase the number of RT-induced antigen-specific CTLs. Co-administration of an HER2-specific mAb with an HER2-targeted tumor vaccine enhanced the induction of HER2/neu-specific CD8-positive T cells through Fc-mediated dendritic cell activation [27]. Irradiation of HER2-expressing tumors showed vaccine-like effects, suggesting that treatment with an anti-HER2 mAb combined with irradiation can induce HER2-specific CTLs. Besides the anti-HER2 mAb, an agonistic anti-OX40 mAb that intensively enhances T-cell activation and proliferation is a candidate combination drug [28]. Previously, we have shown that an anti-OX40 mAb can abrogate regulatory T-cell-mediated suppression [29]. An anti-OX40 mAb causes tumor shrinkage outside the irradiated field in other tumors [30, 31], and further synergistic effects are expected when combined with ICIs.

This study has some limitations. First, while this study describes the importance of tumor antigen-specific CD8-positive T cells in the RT-induced antitumor immune effect, it does not show that the antitumor effect is abrogated when CD8-positive T cells are inhibited. Second, immunological experiments were conducted on the HER2 antigen, a foreign antigen for FVB mice. Therefore, the immune environment differs from that in patients with HER2-expressing tumors, who are tolerant of this endogenous tumor antigen. In the future, a tolerance model, such as the HER2-transgenic FVB mouse model that is tolerant to the HER2 antigen and causes spontaneous HER2-overexpressing breast cancer and then metastases, should be employed to generate clinically relevant result. Third, we showed the HER2-specific CTLs induction by the combination of RT and ICIs using only an HER2-overexpressing tumor cell line. However, to obtain convincing evidence of RT-induced antitumor immune effects on HER2-expressing tumors, it is necessary to compare these results with those of a non-HER2-overexpressing cell line or another HER2-overexpressing cell line. Fourth, we have not examined the optimal timing of ICIs administration. In particular, the effective timing of the administration of the anti-CTLA4 mAb, which may deplete Tregs and prevent local Treg-accumulation of the tumor, needs to be investigated in detail.

In conclusion, RT induces an antitumor immune effect in HER2-positive tumor-bearing mice. Moreover, combining the anti-PD-1 mAb and anti-CTLA-4 mAb administration enhanced the RT-induced distant antitumor effects involved in the accumulation of HER2-tumor antigen-specific CD8-positive T cells at HER2-positive tumor sites. However, FOXP3-positive Tregs with immune inhibitory functions also accumulated at the tumor sites. Our data suggest that RT with dual ICI may be a promising strategic option for the treatment of distant metastatic HER2-positive tumors; however, optimizing the timing of dual ICI or the combination of drugs inhibiting the Treg function is further required to enhance the RT-induced durable antitumor immune effects.

## Abbreviations

Ab: Antibody
CTL: Cytotoxic T-lymphocyte
HER2: Human epidermal growth factor receptor 2
ICI: Immune checkpoint inhibitor
IHC: Immunohistochemistry
ICS: Intracellular cytokine staining
mAb: Monoclonal antibody
RT: Radiation therapy
Treg: Regulatory T cell
SD: Standard deviation
TIL: Tumor-infiltrating lymphocyte
